# Prioritizing ‘equity’ in COVID-19 vaccine distribution through Global Health Diplomacy

**DOI:** 10.34172/hpp.2021.36

**Published:** 2021-08-18

**Authors:** Bawa Singh, Vijay Kumar Chattu

**Affiliations:** ^1^Department of South and Central Asian Studies, School of International Studies, Central University of Punjab, Bathinda, India; ^2^Department of Medicine, Temerty Faculty of Medicine, University of Toronto, Toronto, ON M5G 2C4, Canada; ^3^Institute of International Relations, The University of the West Indies, St. Augustine, Trinidad and Tobago; ^4^Department of Public Health, Saveetha Medical College, SIMATS, Saveetha University, TN, Chennai, India

**Keywords:** Equity, Vaccine, Health diplomacy, Global health, Pandemic, Social determinants

## Abstract

With over 4 million deaths worldwide, the current coronavirus disease 2019 (COVID-19)pandemic is regarded as one of the worst pandemics in history. With its wider devastating consequences, even so-called affluent countries could not provide full coverage for COVID-19vaccines and medications to all of their citizens. Against this backdrop, the main aim of this article is to examine how Global Health Diplomacy (GHD) can play a role in prioritizing vaccine equity in the global health agenda in the fight against COVID-19. The majority of developed countries’ healthcare systems have been exposed and have reached a tipping point.After the completion of eighteen months of the pandemic, only five countries were able to produce vaccines for the treatment of COVID-19. This pandemic has divided the world into two blocs: those with vaccines, such as the United States, the United Kingdom, Russia, China, and India; and those without, such as the rest of the world. The greatest challenges are vaccine inequalities, inequities and distribution, which undermine the global economic recovery. Many poor countries are still waiting for the initial doses to be delivered to their citizens, while some rich nations are planning for booster doses. GHD plays a critical role in establishing successful global collaborations, funding mechanisms and ensuring international cooperation through the combined efforts of all stakeholders. Besides, global solidarity is necessary to lessen the wider gaps between the vaccination status of rich and poor nations. Therefore, through GHD, the vaccine gaps and inequities can be addressed to strengthen global health security and accelerate global economic recovery.

*“Vaccine inequity is the world’s biggest obstacle to ending this pandemic and recovering from COVID-19”*

**Dr Tedros Adhanom Ghebreyesus, Director-General, WHO**

## Introduction

The outbreak of the coronavirus disease 2019 (COVID-19)pandemic has been one of the most tragic events of the 21^st^ century. It is one of the ongoing coronavirus diseases caused by the severe acute respiratory syndrome coronavirus 2 (SARS-CoV-2), which was identified for the first time in Wuhan, China.^1^ Given its massive destruction in terms of men and material, the World Health Organization (WHO) declared it as a Public health emergency of international concern on 28 January 2020 and pandemic on 11 March 2020.^2^ As of 21 July 2021, there were more than 191 million cases and around 4.1 million deaths due to the COVID-19pandemic.^3^ As of 20 July 2021, globally, a total of 3.6 billion (approximately) vaccine doses have been administered.^3^ The concept of Global Health Diplomacy (GHD) has been gaining great momentum since the pandemic has started. Many issues, ranging from accessibility, affordability, and equity of healthcare facilities, have been raised by developed and developing nations to address the challenges posed by the pandemic on their health systems. Kickbusch et al have defined GHD as the multilevel and multi actors’ negotiation processes used to save and manage the global health policy environment for health.^4^ On the other hand, Fauci had defined the same as “winning the hearts and minds of people in poor countries by exporting healthcare, expertise, and personnel to help those who need it most.^5^ Further, Fidler has defined GHD as “Policy shaping processes in which state, non-state, and other institutional actors negotiate responses to health issues or use health principles or framework in policy shaping and negotiation techniques, to achieve other political, economic and social goals.^6^

GHD has become an important determining factor for foreign policy. The foreign policy of any country has objectives to be achieved in terms of national, international, and geopolitical interests. Health diplomacy used to become an important element for achieving such interests. As per WHO EMRO,^7^ the Health Diplomacy is used to (1) enhance health protection and public health; (2) improved relations between states and a commitment by a wide range of actors to work together to improve health and (3) achievement of fair outcomes that support the goals of poverty reduction and equity. As per Kickbusch and Kökény,^8^ the four factors had contributed to the acceptance and universalization of GHD. Firstly, because of its soft power importance which is an important instrument for improving bilateral and multilateral relations among nation-states. Secondly, the realm of health diplomacy is expanding, and many new actors, including WHO, are shaping the global policy environment in the sphere of health and its determinants. Thirdly, globalization accelerated the rise of cooperation between developed and developing countries and heightened the need for health diplomacy, leading to binding and non-binding agreements. Fourthly, health diplomats’ role in making agreements and negotiations is relevant.

Some scholars^9^ have argued that GHD can be pursued in the forms of disease identification and prevention and responding to health issues. Health diplomacy used to become an instrument of providing medical healthcare facilities during health emergencies. In the globalized world order, the health challenges have no longer been treated at the local level, and rather the same is required as the international response. In the recent past, during the epidemics of H5N1 in 2007, H1N1 in 2009, Ebola^10^ in 2014, Zika^11^ in 2016, and currently, during this COVID-19 pandemic,^12^ health diplomacy has recorded some success stories. Notwithstanding the divergent motivations and goals, GHD has been pursued by several countries globally and has become a success story in the recent past.

### The healthcare divide across the world

The healthcare facilities, particularly in the developing and least developed countries, have been drastically underfunded. The vast majority of these countries have not been able to provide basic healthcare facilities to their citizens. Moreover, these countries have been characterized by the lack of doctors, paramedical staff, medical infrastructures, vaccines, medicines, and even if the vaccines/medicines available that is/are at exorbitant prices. As per the report of the WHO, about 22 countries wherein the entire healthcare systems need to be rebuilt.^13^

As per the findings of the WHO and World Bank (WB) (2017) reports,^14^ more than half of the world’s population have not been in the position of obtaining the essential healthcare facilities. Rather, several households are being driven into the abject poverty trap given their out-of-pocket healthcare services expenditure. Razavi has demonstrated that in the present context, about 800 million people used to spend more than 10% of their household budgets on healthcare expenditure for themselves.^15^ Therefore, it is very difficult for 930 million people to manage their healthcare expenditure.^16^ According to World Bank, about 150 million would be likely to be pushed into extreme poverty given the healthcare expenditure, which is enough higher to push them into the poverty trap along with forcing them to survive on less than US$1.90 per day.^17^

Given the poor healthcare facilities, abject poverty, inadequate research and development funds, the accessibility, affordability, and equity of healthcare facilities have become a critical challenge, particularly for the low-income countries in the context of COVID-19. The vaccines have been produced by the developed countries, particularly by China, the US, UK, Russia, and India. At the same time, it is important to point out that the distribution of the same among the poor countries had not taken place. Meanwhile, nearly three fourth of the COVID-19 vaccine doses have been administered only by the rich ten countries globally, accounting for roughly 60% of the global GDP as per the WHO.^18^ It has been reported that the rich countries had stored the bulk of COVID-19 vaccines.

In contrast, the frontline health workers and people of the developing countries/regions have been dying due to COVID-19 due to the disparities because of inequities, inequalities, issues related to affordability, accessibility of vaccines, and healthcare facilities. The health workers have been exposed to the risk of a pandemic, particularly in the developing countries from the Asian, African and Latin American, and Caribbean countries. About 2.5 billion people, particularly from the 130 developing and least developed countries, are yet to even administer a single dose.^18^ However, the COVID-19 vaccine has been rolled out in rich and middle-income countries, which have prioritized their people and health care workers, whereas, on the other hand, none of the Sub-Saharan African countries received the same.^19^

Realizing the inability of the developing countries for accessibility and affordability of the COVID-19 vaccine, the WHO started the program of COVAX, primarily focusing on the equity of vaccines. The primary focus of this initiative was to roll out COVID-19 vaccines for all countries in general and in 92 lower-income countries in particular within the 100 days of 2021. Moreover, 20 percent of the population of all countries will be inoculated by the end of the year.^20^ The UN Secretary-General Antonio Guterres sharply criticized the “wildly uneven and unfair” distribution of COVID-19 vaccines, saying ten countries have administered 75% of all vaccinations and demanding a global effort to get all people in every nation vaccinated as soon as possible. The UN General Secretary Anthony Guterres conveyed to the higher-level meeting of the UN Security Council that about 130 countries had not received even a single dose of the COVID-19 vaccine. He declared that “at this critical moment, vaccine equity is the biggest moral test before the global community” and had called for an urgent global resolution plan to ensure equitable vaccine for all.^21^

It becomes clear that some countries have been making efforts to vaccinate their entire population. At the same time, several other countries have nothing to offer their citizens. It provides a false sense of health security. Against this background, the WHO Chief Tedros has argued that “the inequitable distribution of vaccines is not just moral outrage. It’s also economically and epidemiologically self-defeating.” As of 15 April 2021, more than 660 million people have been vaccinated across the world. However, the major concern for humanity is the heightening of the gap between the rich and poor countries, which is evident from the fact that about 86% of doses have already been administered in high and upper-middle-income countries. In contrast, a growing concern is that low-income countries have been able to administer only 0.1 %. As of 15 April 2021, only 33 million COVAX doses were received by 70 countries.^22^

### Social and geopolitical determinants of health

As per the study of some scholars,^23^ the social determinants of health have been defined as those economic and social conditions in which individuals and the group/s have been differentiated in terms of health status differences. Moreover, the social determinants for those health promotive factors in which once living and working conditions rather than the risk factors influence disease or the only ability to disease or injury. The distributions of social determinants can be shaped or reshaped by the public policy reflecting the prevailing political ideology of the particular country or region.^24^

In the present context, particularly when the pandemic was at its peak, the world has been divided into rich and poor countries. This division has been characterized by health inequalities. The researchers^25^ had emphasized that globalization has drastically impacted the social determinants in terms of healthcare facilities. The inferences can substantiate the argument mentioned above posited that globalization has created drastic unequal impacts on various facets of life and resulted in uneven distribution of power and wealth both within and across the national boundaries, seriously impacting the healthcare facilities.^25^ The Organisation for Economic Cooperation and Development (OECD) has highlighted the major differences even in the developed countries in terms of healthcare status indicators such as infant mortality, life expectancy, the incidence of disease, death from injuries, etc. The WHO stated that “This unequal distribution of health-damaging experiences is not in any sense a ‘natural’ phenomenon but is the result of a toxic combination of poor social policies, unfair economic arrangements [where the already well-off and healthy become even richer and the poor who are already more likely to become even poorer], and bad politics.”^26^

Scholars have argued that evaluating and understanding the national health and health inequalities determinants could become an important guide policy for global healthcare equity, accessibility, and affordability. Of course, one approach to understanding the social determinants of health care issues, wherein the geopolitical perspective, has been figuring very prominently. As per this framework, the important elements of social determinants include the population, healthcare facilities, job security, housing, transportation, poverty, and the most important health inequalities in the population.^27^ On the other hand, commercial and corporate factors have also affected health behaviors such as smoking and consuming those food products heavily loaded with sugar contents.^28^ Some studies have also shown that some diverse social factors affect health outcomes and identify those contexts as the eco-social determinants of health status.^29^

The geopolitical determinants consist of governments, public policies and interests of the countries, geographies, and most importantly, the bilateral, regional, and multilateral engagements in terms of international relations.^30^ The main purpose of understanding geopolitical determinants in this context is to identify the individual health outcomes as products of national policy at the level of local or original levels. This understanding may also help recognize and identify those policies that are being influenced by the geographical factors, leadership, and most importantly, bilateral, regional, or multilateral relations with the neighboring countries and distribution of required healthcare resources. Therefore, the accounts of geopolitical factors and issues used to become one of the very critical approaches to understanding the healthcare issues, concerns, and determinants. To understand this augment and perhaps perspective, the example of migration would be very helpful in this context.^31^ As migration is being shaped by geopolitical factors like civil war, ethnic conflict, war, colonization, climate change, and treatment of minorities, it has been considered an important element and determinant of an individual’s mental health.^32^ Understanding the geopolitical determinants and quoting the example of migration highlights the importance of understanding healthcare issues. As the migrants intersect with the local population, many social determinants such as employment, education, and social support, put them in a conflictual situation, which drives into chronic stress affecting their health.

In social and geographical determinants, violence is another important key health determinant.^33^ Therefore, identifying and recognizing state violence and physical and sexual interpersonal violence should also be considered potential key indicators for strengthening the healthcare systems. Against this background, the prevention and response towards the violent conflicts between and within countries would have drastic and positive impacts on violence reduction, public health, and public mental health policies.

### ‘Healthcare for all’ through Global Health Diplomacy

Given the outbreak of COVID-19, the scope of GHD has been increased exponentially. More avenues have been opened given the establishment of immunity division and vaccine division of the international organizations. The GHD refers to a system wherein the organization’s communication and negotiation processes shape the global health policy environment in general and in the context of health and its determinants in particular in the prevailing scenario. Health has become an important part of GHD at WHO, United Nations, Organization for Economic Cooperation and Development (OECD), G-7, G-20, the European Union, and the BRICS group (Brazil, Russia, India, China, South Africa). The Organisation of Islamic Countries (OIC) has been recently established an organization that is supposed to look after the healthcare issues of the member countries. The GHD has remained very proactive in the context of the human immunodeficiency virus (HIV), Maternal and child health issues, communicable and noncommunicable diseases (NCDs) in general, and particularly in the current COVID-19 pandemic. The inter-governmental, governmental and non-governmental organizations, have played a significant role during the current ongoing pandemic.

Globalization has given a new dimension to donor-recipient relations. Against this background, new kinds of healthcare cooperation between the low and middle-income countries had become part and parcel of the GHD. Simultaneously, the long-term negotiation processes regarding binding and non-binding agreements have been taking place among the rich and poor countries. One of the most noteworthy examples in this area is the World Health Organization’s approval of the Pandemic Influenza Preparedness Framework in 2010. It has been perceived as one of the best global health governance milestones in healthcare issues.^34^ The United Nations High-level Meeting on NCDs in 2011 adopted the political declaration on preventing and controlling NCDs.^35^ The illicit trade of tobacco has very serious impacts not only on an individual’s health, rather the revenue of governments and criminal activities, etc. Therefore, an international treaty, the Protocol to Eliminate Illicit Trade in Tobacco Products, to eliminate all forms of illicit trade was put in place as a global solution to global socio-economic and healthcare problems.

Health has been one of the important elements of Sustainable Development Goals (SDGs). The SDG Goal 3 is one of the 17 important goals which has targeted Good Health and Well-being. SDG 3 has also called for innovation and high-level research and development in healthcare issues through public policy efforts. The goal has also expressed its commitment to provide a renewed focus on mental health issues as well. The declaration by UN Human Rights Council^36^ emphasized “peace, development, health, security and human rights are interlinked and mutually reinforcing,” which is highlighted in the UN SDGs as the interrelated dimensions of “peace, health, and social development.” In this context, Chattu et al have highlighted the role of GHD as a tool of peace^37^ and often functions as a dynamic wheel engaging with these dimensions impacted by the challenges posed by natural disasters, economic crises, and conflicts.^38^

GHD can be a bridge for international cooperation for tackling public health crises, strengthening health systems through emphasizing universal health coverage for sustainable and equitable development, and rebuilding multilateral organizations.^39^ Therefore, if health is truly viewed as a human right, a whole of society and government approach must realize this. Besides, the member states of WHO should put a whole-of-society and government approach to develop national action plans to include attention for the vulnerable groups.^40^ Hence, the priorities of all the countries should be to find the areas of common interest, common operational overlap on development issues, and resource allocation for this global fight against COVID-19.^39^ A recent article by Taghizade et al have summarized the emerging scope of GHD in this post-pandemic world by influencing the five global arenas, namely (1) international cooperation and global solidarity, (2) Global Economy, Trade and Development, (3) Global Health Security, (4) Strengthening health systems and (5) Addressing inequities to achieve the global health targets.^41^

To control and combat the COVID-19 pandemic, the WHO has played a pivotal role through several initiatives. WHO launched COVID-19 Solidarity Response Fund on 13 March 2020 to contain and combat the COVID-19 pandemic. While the main focus of WHO is to work with its partners, the fund would be utilized to track and treat the patients, accelerate the vaccine R&D and provide treatments for all and provide essential supplies and information to frontline workers.^42^ Extending its cooperation and contribution, the UN and WHO and World Food Programme, the COVID-19 Supply Chain Taskforce, were launched in early April 2020. It was tasked to scale up the procurement and provision of testing and diagnostics supplies, biomedical equipment, and personal protective equipment.^43^ The Supply Chain Taskforce had facilitated a shipment of WHO medical cargo from Dubai to Addis Ababa by mid-April to tackle the problem head-on. These medical supplies were transported to several parts of Africa. Paul Molinaro,Chief of Operations Supoort and Logistics at the WHO has argued that if these timely intervention measures had not taken place, about 300 000 to 3.3 million Africans would have died. The WHO has also managed to provide millions of personal protective gear to about 133 countries and diagnostic kits to 126 countries.^44^

The Global Alliance for Vaccines and Immunization (GAVI), established in 2000, has WHO, UNICEF, WB, Bill and Melinda Gates Foundation, along with vaccine industries as major partners. The major focus of the GAVI is to promote the immunization of children from poor countries along with removing the commercial concerns of the vaccine manufacturers. As a response to the COVID-19 pandemic, the GAVI in September 2020 committed to ensuring that any new COVID-19 vaccine would be shared equally between the world’s rich and poor countries. It had also announced the approval of US$ 150 million to help approximately 92 low and middle-income countries for the delivery of future COVID-19 vaccines along with the provision of technical assistance, support, and cold chain equipment.^45^

The Access to COVID-19 Tools Accelerator (ACT Accelerator or ACT-A) has been an initiative of G20, which was announced on 24 April 2020. Concerning the ongoing pandemic, the major focus is to accelerate production and equitable access to COVID-19 diagnostics, therapeutics, and vaccines. During the virtual meeting, the program’s leadership had committed and pledged to provide 2 billion COVID-19 vaccines, 245 million treatments, and 500 tests to control and combat the pandemic. Up to 15 April 2021, it has been reported that US$ 2.7 billion had been received, just one-tenth of the urgent requirement. The program has engaged with more than 170 countries for the COVID-19 vaccine facility.^46^ In those lines of health for all, health equity, affordability, and accessibility, the GAVI has been pursuing the principle- “No one is safe until everyone is safe.” ^47^

With the outbreak of the pandemic, it is expected that humanity would prevail, and the principle that no one is safe until everyone is safe would be put into practice through the equal distribution of the vaccine. However, the irony is that market forces used to prevail, and therefore rich countries not only ensured vaccination of their citizens rather reserved the same more than needed. Meanwhile, the majority of the poor countries had left without vaccines. Against this background, the role of the GHD becomes very critical to resolving the healthcare inequity. In the present context, GHD, on the part of WHO, along with several inter-governmental, governmental, and non-governmental organizations, has been playing a very significant role in providing healthcare equity.

Notwithstanding the pro-active role of the GHD, there is still a wide gap between the vaccination status of rich and poor countries. If the vaccination gap between the rich and poor countries is not removed, then the rich countries wouldn’t be safe. Therefore, through its GHD, WHO could bring all countries on one board to convince them that no one is safe until everyone is safe. Safety would come through vaccination equity.

## Conclusion

Therefore, the global health governance mechanisms committed to eradicating diseases and addressing the challenges of new and emerging health issues should also prioritize the health disparities in the global south on the inequalities and inequities and other aspects of accessibility and affordability. The role of GHD is pivotal in creating successful global partnerships and funding mechanisms to ensure cooperation, show solidarity and ensure collective efforts with the full participation of all the actors. The global SDGs can only be achieved through active participation of all the key players of global-south and developed nations when ‘Health For All’ is perceived as a common shared goal of everyone. Amid this pandemic crisis, there is an urgent need to lessen the wider gaps between the vaccination status of rich and poor nations to promote equity, accessibility, and global health security.

## Acknowledgements

The authors thank the reviewers for their critical feedback in improving the quality of this manuscript.

## Funding

Nil.

## Competing Interests

Vijay Kumar Chattu is an Editorial Board of Health Promotion Perspectives. Other authors declare that there is no conflict of interest.

## Ethical approval

Not applicable.

## Authors’ contributions

BW and VKC are involved in the concept, design, literature review, and preparation of the initial draft. VKC edited and finalized the draft addressing the critical aspects. Both the authors have approved the final version before submission.

## Disclaimer

The views expressed in this publication are those of the authors based on their experience and the available evidence. Further, the authors claim that no part of this paper is copied from other sources.
